# Chinese Herbs Interfering with Cancer Reprogramming Metabolism

**DOI:** 10.1155/2016/9282813

**Published:** 2016-05-05

**Authors:** Zhangfeng Zhong, William W. Qiang, Wen Tan, Haotian Zhang, Shengpeng Wang, Chunming Wang, Wenan Qiang, Yitao Wang

**Affiliations:** ^1^Institute of Chinese Medical Sciences, State Key Laboratory of Quality Research in Chinese Medicine, University of Macau, Macau; ^2^Yale University, New Haven, CT 06511, USA; ^3^School of Pharmacy, Lanzhou University, Lanzhou, Gansu 730000, China; ^4^Division of Reproductive Science in Medicine, Department of Obstetrics and Gynecology, Feinberg School of Medicine at Northwestern University, Chicago, IL 60611, USA

## Abstract

Emerging evidence promotes a reassessment of metabolic reprogramming regulation in cancer research. Although there exists a long history of Chinese herbs applied in cancer treatment, few reports have addressed the effects of Chinese herbal components on metabolic reprogramming, which is a central cancer hallmark involved in the slowing or prevention of chemoresistance in cancer cells. In this review, we have focused on four core elements altered by metabolic reprogramming in cancer cells. These include glucose transport, glycolysis, mitochondrial oxidative phosphorylation, and fatty acid synthesis. With this focus, we have summarized recent advances in metabolic reprogramming of cancer cells in response to specific Chinese herbal components. We propose that exploring Chinese herbal interference in cancer metabolic reprogramming might identify new therapeutic targets for cancer and more ways in which to approach metabolism-related diseases.

## 1. Introduction

Since the 1920s, cancer cell metabolism has been studied in the context of the so-called Warburg effect. This metabolic feature of neoplastic cells involves many complex biochemical reactions and multiple interrelated signaling pathways, which have yet to be fully elucidated. The Warburg effect is an ubiquitous event in the malignant proliferation of cancer cells, distinguishing them from normal cells, and recently a growing number of novel findings promoted a reassessment of metabolic reprogramming regulation in cancer research [1]. For example, recent work suggests that the Warburg effect regulates chemosensitivity in Taxol-resistant human breast cancer cells [2]. Other studies have shown that it influences estrogen-related receptor expression [3]. In autophagy-senescent fibroblasts that promote the growth and metastasis of human breast cancer cells, the Warburg effect participates in the crosstalk between fibroblasts and cancer cells. The precise relationship between the two cell types is governed by mitochondrial metabolism [4].

Many reviews and research studies show that systemic metabolic disorders intersect with cancer development, and metabolomics profiling for prognostic and predictive markers has identified shared markers associated with both conditions [5–9]. Recent work with several tumor types has documented the synergistic effect of combining 2-deoxy-D-glucose (2-DG) and berberine, two tumor cell-toxic compounds, to target glycolysis and oxidative phosphorylation (OXPHOS) simultaneously [10]. In nuclear magnetic resonance- (NMR-) based metabonomics research, significant differences for prognosis have been documented for acute myeloid leukemia (AML) patients with “favorable” vis-à-vis “intermediate” statuses in the cytogenetic determinants governing glycolysis, the tricarboxylic acid (TCA) cycle, and fatty acid synthesis [11].

The metabolic phenotype of cancer cells provides a great deal of valuable information about treatment design and prognosis. This information overrides genetic and individual diversity as well as pathologic diversity of specific cancer cell types. For example, in human colorectal cancer, the presence or absence of significantly altered metabolites correlates with recurrence and survival rates after surgery and chemotherapy [12].

Large numbers of studies identifying these correlative effects suggest that exogenous metabolic regulation has good therapeutic potential for cancer treatment. Across many published studies, this has been shown to be particularly true when related to targeting the uptake and the utilization of glucose, the abnormally enhanced glycolysis, altered mitochondrial oxidative phosphorylation (OXPHOS), and deregulated fatty acid synthesis. Meanwhile, other reprogramming events have been shown to occur in parallel with the ones identified above, including altered glycine consumption and activated mitochondrial glycine biosynthetic pathway [13]. Finally, many studies have focused on the systems biology and metabolic transformation of cancer cells, where the key enzymes in glycolysis, the TCA cycle, and the pentose phosphate pathways (PPP) were investigated as correlated metabolic changes occurring within the context of the Warburg effect [14].

In Chinese medicine, treatment strategies have emphasized promoting blood circulation, supporting health and energy while strengthening body resistance, heat clearance, and systemic detoxification, resolving phlegm production, promoting T cell cytotoxic functions, dispersing edema, and relieving pain. In addition, many natural products derived from Chinese herbs have been shown to have therapeutic potential toward the prevention of cancer initiation and progression [12, 15]. One example is the Chinese botanical agent berberine, which inhibits mitochondrial complex I and interacts with the adenine nucleotide translocator in cancer cells [16]. The powdered fruiting bodies of* Pleurotus eryngii* (DC. ex Fr.) Quel have been shown to significantly inhibit the proliferation of several cancer cell lines (A549, BGC-823, HepG2, and HGC-27) and to have immunopotentiation activity in RAW 264.7 cells [17]. 1,2,3,4,6-Penta-O-galloyl-beta-D-glucose, which is a polyphenolic compound isolated from* Rhus chinensis* Mill, regulates a series of metabolic genes in glycolysis, pyruvate metabolism, gluconeogenesis, and tyrosine metabolism in the breast cancer cell line, MDA-MB-231 [18]. These compounds are isolated examples of an extended body of work, with biochemical agents that have been used in China for up to 3,000 years that recently have shown targeted inhibition of cancer metabolic reprogramming, by which tumor initiation, progression, and spread might be addressed.

In this review, we have focused on glucose, glycolysis, mitochondrial OXPHOS, and fatty acid synthesis and have identified recent successful studies using Chinese herbs that target and inhibit cancer cell growth.

## 2. Glucose Transport

Abnormal glucose metabolism is closely linked with cancer cell metabolism. In normal cells, glucose uptake sustains the two main metabolic processes that produce ATP as an end product, which are glycolysis and mitochondrial OXPHOS [19–24]. There have been few studies on glucose uptake by cancer cells; instead, the vast majority of work has focused on the activity of glucose transporters (GLUTs). In recent years, another family of glucose transporters, called sodium-dependent glucose transporters (SGLTs), was studied for their observed ability to slow tumor cell growth. Some components derived from Chinese herbs exert direct GLUT inhibition. Notably, GLUT expression is significantly different in cancer cells, in relation to their normal counterparts. This property of cancer cells makes them vulnerable to GLUT inhibitors, which reduce glucose consumption [25–28]. Tetrandrine, a bisbenzylisoquinoline alkaloid isolated from* Stephania tetrandra* S Moore, reduces glucose uptake in cancer cells, and this energetic impairment induces apoptosis [29]. Finally, chlorogenic acid, a phenolic secondary metabolite isolated from many Chinese herbs (*Eucommia ulmoides* Oliv. and* Lonicera japonica Thunb.* among others), has significant effects on glucose transport in cancer cells, through activating the AMPK signaling pathway [30].

## 3. Glycolysis

Cancer cells require high levels of glycolytic intermediates to support their biosynthetic requirements [64]. Many promising anticancer agents, currently under study, are compounds that inhibit aerobic glycolysis [65–67]. Some of these agents also target autophagy in cancer cells, suggesting that they might be used for combinatorial therapies [68].

Some bioactive compounds derived from Chinese herbs are now known to regulate glycolysis [67]. For example, prosapogenin A, isolated from* Veratrum*, regulates the expression of glycolysis-related genes to induce apoptosis in human cervical carcinoma (HeLa), hepatocellular carcinoma (HepG2), and breast adenocarcinoma (MCF-7) cells [45]. Other Chinese herbal components target different glycolytic intermediates.

Epigallocatechin, for example, is one component derived from the Chinese herb* Spatholobus suberectus* that targets glycolytic lactate production by inhibiting the expression or activity of lactate dehydrogenase A (LDH-A), an important enzyme checkpoint in glycolysis that catalyzes the interconversion of pyruvate and L-lactate. Its activity has been shown to induce apoptosis and suppress breast cancer growth* in vivo*, as upregulated LDH-A facilitates glycolysis and reduces tumor dependency on oxygen [69]. LDH-A promotes tumor initiation, progression, and metastasis, and it is a prognostic factor for poor survival [70]. Inhibiting LDH-A activity significantly reduces cell proliferation and tumor size, and it induces elevated intracellular oxidative stress, resulting in apoptosis. Silencing LDH-A also contributes to suppressing tumorigenicity in breast cancer cells [71]. There is also evidence that the LDH-B isozyme also participates in tumor development and is regulated by oncogenic transcription factors mammalian target of rapamycin (mTOR) and signal transducer and activator of transcription 3 (STAT3) [72].

Oleanolic acid, another Chinese herbal derivative, suppresses aerobic glycolysis via the PK-M2/PK-M1 switch that accompanies mTOR/c-Myc/heterogeneous nuclear ribonucleoprotein (hnRNP) signaling regulation [39]. Pyruvate kinase isoenzyme type M2 (PK-M2), like LDH-A, is a critical enzyme in glycolysis, and its underlying mechanisms have been also explored extensively in the context of tumor cell physiology [39, 73]. In hepatocellular carcinoma cells, targeting PK-M2 shows therapeutic potential, as it regulates epithelial-mesenchymal transition and migration [74]. Of interest, PK-M2 activity is augmented by hypoxia-inducible factor 1*α*- (HIF-1*α*-) mediated transcription activation in mTOR-hyperactive cancer cells [75].

Finally, neoalbaconol, an isolate of the fruiting body of* Albatrellus confluens*, induces energy depletion in cancer cells by inhibiting the phosphatidylinositol 3-kinase (PI3K)/HK2 pathway and reduces glucose consumption and ATP generation [38]. Hexokinase 2 (HK2) is also a key metabolic regulator implied in many tumor types.

## 4. Mitochondrial Oxidative Phosphorylation (OXPHOS)

Mitochondrial OXPHOS is abnormal in cancer cells, and many studies suggest that it may underlie tumor initiation, growth, and metastasis of cancer cells. However, until recently it has been unclear (1) whether these abnormalities are the results of mitochondrial dysfunction and (2) whether mitochondrial functional suppression would inhibit tumor cell growth [76–79]. Many current studies use natural origin derivatives to target mitochondrial OXPHOS in tumors and to study the exerted synergistic effects these compounds have in the presence of other chemotherapeutic agents [80]. For example, berberine, as mentioned previously, synergistically enhances the suppression of cancer cell proliferation through ATP depletion, when it is combined with 2-deoxyglucose (2-DG) [10].

Other natural origin derivatives may act alone or use other mechanisms to achieve similar outcomes. Chrysophanol, an anthraquinone derivative, induces necrotic tumor cell death in Hep3B hepatoma cells by decreasing ATP levels [32]. Shikonin, a major bioactive component isolated from* Lithospermum erythrorhizon*, induces apoptosis through reactive oxygen species (ROS) deregulation and OXPHOS uncoupling [48]. Of interest, shikonin seems to accumulate preferentially in the mitochondria, and its induction of ROS species results in the collapse intracellular redox balance [49]. A botanical extract obtained from the leaves of the tropical Papaya plant* Carica papaya* exerts a significant cytotoxic effect on hypoxic cancer cells, by way of hypoxia-inducible factor (HIF) inhibition [81]. HIF transcription factors regulate glucose and lipid metabolism, by mediating the switch from oxidative phosphorylation to glycolysis (HIF-1) and coordinating *β*-oxidation of fatty acids to maintain cell survival (HIF-2) [82]. More importantly, they regulate cell response to hypoxic environments and adapt cancer cell metabolism to promote cell survival when faced with the unreliable oxygen supply of the tumor microenvironment. HIF has become an attractive target for cancer therapy as HIF regulation is correlated with glucose metabolism and cell survival in many cancer cells, including HepG2 hepatoma cells [81, 83–85]. Finally, vitamin C is cytotoxic in HIF-positive cancer cells, as HIF inhibition by* Carica papaya* extract contributes synergistically in these cells with vitamin C to reduce ATP regeneration [86].

## 5. Fatty Acid Synthesis

Because they depend upon glycolysis for energy, cancer cells generally contain more glycolytic metabolites than their normal cell counterparts. This surfeit of nucleotide and protein precursors combined with lipids produced in fatty acid synthesis act to promote tumor initiation, proliferation, and spread [87]. Fatty acid synthesis provides macromolecule support for tumor cell proliferation and is activated above control cell levels in many tumor types [88, 89]. Many components of Chinese herbs have fatty acid synthase (FASN) inhibitory activity [90, 91]. In the human hepatoma cell line HepG2, botanical compound quercetin induces apoptosis through inhibiting the activity and expression of intracellular FASN [46]. Osthole, isolated from* Cnidium monnieri* (L.) Cusson, inhibits FASN, and by this activity it induces apoptosis in human epidermal growth factor receptor 2- (HER2-) overexpressing breast cells [44]. Oridonin, a diterpenoid isolated from* Rabdosia rubescens*, exerts antitumor effects by inhibiting fatty acid synthesis in two human colorectal cancer cell lines, SW480 and SW620 [42].

The studies cited above are selected from a large number of recent reports which identify FASN inhibitors, from Chinese herbal components, as being effective agents for metabolic conditions that do not target the propagation of neoplasia directly, but through their ability to promote blood circulation, prevent blood stasis and pathological coagulation, support systemic energy demands, and strengthen resistance to microbes and clear toxic materials [92].

## 6. Discussion

### 6.1. Chinese Herbs Interfere with Cancer Cell Metabolism through Many Pathways and Targets

We have shown previously that berberine, an alkaloid isolated from Chinese herbs, interferes with cancer cell metabolism through many different pathways and targets, including but not limited to glycolysis (PKM2, PFKP) and fatty acid synthesis (ACC, ACL) [31, 93, 94]. Other herbal derivatives also interfere with cancer cell metabolism in different ways, and their activities are summarized in Table 1. When compared to conventional approaches (surgery, radiation, and chemotherapeutic drug agents), agents like berberine are more tolerable and less toxic than these listed procedures whose use is the standard of care for cancer in Western medicine. Recent studies of Chinese medicine-affiliated compounds, examined through the lens of modern medical practices, have shown that they are sustainable and progressive modes of antitumor activity. These findings should be interpreted against a large body of data (centuries of use of these agents) showing that their systemic cytotoxicity is low or absent [15].

Worldwide interest in Chinese herbs has been reawakened in light of the realization that metabolic reprogramming is central to the biology and survival of cancer cells. Metabolic reprogramming is adaptive for tumor cells, but in a limited context: its requirements must be met or tumor cells become susceptible to intracellular or host-cytotoxic mechanisms that can kill them. This identifies a large new set of potential therapeutic targets in tumor cells, and many of these targets are specifically affected by Chinese herbal components. The aforementioned aspects of glycolytic inhibition can be addressed individually for cancer treatment. However, unilateral regulation of OXPHOS or glycolysis through inhibition alone might not completely address the complex energy status changes that must be sustained in cancer cells [95]. Instead, whole-network interference, a concept to be explored in future systems biology studies, is likely to be much more effective.

We propose here that Chinese herbs and herbal components with documented antitumor activity may fill this deficiency, in that many of these agents address multiple targets in the glycolytic “Achilles heel” (e.g., the Warburg effect).

### 6.2. Perturbation of Cancer Metabolism with Chinese Herbs Disrupts Neoplastic Homeostasis

A consensus hypothesis regarding antitumor Chinese herbs is that perturbation of cancer metabolic reprogramming (the reduction of available metabolic energy for the cells) essentially amounts to a major disruption of a metabolic state that has been transformed, by the neoplastic process, into a new kind of homeostasis, which maintains cancer cell viability and proliferation. In the studies cited above, a common aspect of Chinese herbal component antitumor effects is their ability to break down glycolytic reprogramming in cancer cells, thereby exposing them to toxic agents and mechanisms that otherwise would be ineffective. This idea has support from many studies; for example, melanoma cells can be reprogrammed, by PB transposons, to return to differentiation, normal proliferation, and low metastatic potential [96].

In addition to their ability to disrupt glycolytic metabolism in cancer cells, Chinese herbs restore a normal homeostatic state, both in cells and systemically, and together these effects promote health and recovery in cancer-affected individuals. Finally, as noted above, thousands of years of experience support safe application in the clinic. Therefore, what we will now call “perturbation regulation” (restoration of the new metabolic homeostasis established by neoplastic transformation) by Chinese herbs is reasonable and forward-looking [15, 92, 97]. Although the exact metabolic regulatory pathways and the definite mechanisms addressed by many of these agents are yet unknown, it is now time to fill these information gaps.

### 6.3. Chinese Herbs and Herbal Derivatives Are Promising Therapeutics for Cancer and Other Metabolism-Related Diseases

Targeting glycolytic tumor reprogramming may be useful in combination with current treatments of cancer, for example, chemotherapy, by making lower doses of toxic agents more effective. Another promising use for Chinese herbs would be in metabolic syndrome disorders, including diabetes, wherein their ability to perturb pathological homeostatic mechanisms (by perturbation, returning to normal intracellular and systemic metabolism) would offer a gentle and effective treatment protocol for these diseases, whose basic pathophysiology involves harmful metabolic reprogramming that cannot be disrupted by current treatment regimens.

## 7. Conclusion

**Figure 1:**
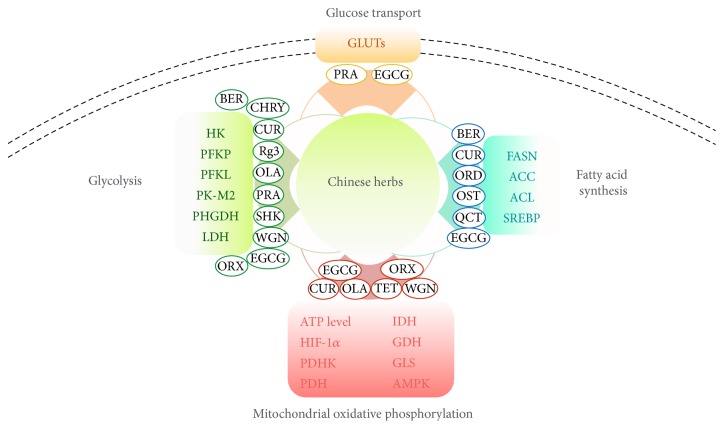
Cancer metabolic reprogramming and Chinese herbal interference. Metabolic reprogramming in cancer cells has four core elements: glucose transport, glycolysis, mitochondrial oxidative phosphorylation, and fatty acid synthesis. Fourteen derivative compounds of Chinese herbs have been shown to interfere with the complex and interrelated biochemical reactions that result in cancer cells achieving that metabolically reprogrammed dysfunctional energy homeostasis. While some of the mechanisms behind the antitumor activity of these chemical agents are known, these compounds have potent metabolic regulatory potential that has yet to be tapped. Herbal derivative abbreviations: BER: berberine; CUR: curcumin; Rg3: ginsenoside 20(S)-Rg3; OLA: oleanolic acid; ORD: oridonin; OST: osthole; PRA: prosapogenin A; QCT: quercetin; SHK: shikonin; TET: tetrandrine; WGN: wogonin; EGCG: epigallocatechin gallate; ORX: oroxylin A; CHRY: Chrysophanol.

In this review, we propose that Chinese herbs and herbal components with identified antitumor efficacy should be studied in detail, toward the identification of multiple new targets and pathways that are expressed in “neoplastic homeostasis.” We have summarized several of the core changes that occur in metabolically reprogrammed cells and used these to illustrate where recent and older work have already given us a new direction (perturbation of the aforementioned neoplastic homeostasis). This new direction provides a novel context for the identification of targets and pathways that expose tumor cells to the toxicity of Chinese herbs. As noted above, many compounds derived from Chinese herbs are known to have potent metabolic regulatory potential, as indicated in Figure 1.

## Figures and Tables

**Table 1:** Bioactive compounds isolated from Chinese herbs interfere with cancer metabolic reprogramming.

Bioactive compounds	Chinese herbs	Cancer model	Metabolic regulation	Potential mechanisms	References
Berberine	*Coptis chinensis* (Huang lian)	Breast cancer MCF-7 cells	Mitochondrial OXPHOS; glycolysis; fatty acid synthesis	Inhibits the phosphorylation of PKM2 and PFKP and regulates ACC and ACL pathways	[31]

Chrysophanol	*Rheum palmatum* (Da huang)	Human liver cancer Hep3B and J5 cells	Mitochondrial OXPHOS; ATP generation	Decreases levels of GST, SOD (Cu), SOD (Mn), and catalase and increases LDH activity	[32, 33]

Curcumin	*Curcuma longa* (Jiang huang)	ESCC *EC109 cell*; human colorectal cancer HCT116 and HT29 cells; human hepatoma HepG2 cells	Glycolysis; fatty acid synthesis	AMPK-dependent metabolic regulation; downregulates the activity of hexokinase II (HKII) and the expression of HKII and FAS	[34–36]

Ginsenoside 20(S)-Rg3	*Panax ginseng* (Ren shen)	Ovarian cancer cells	Glycolysis	Targets the STAT3/HK2 pathway	[37]

Neoalbaconol	*Albatrellus confluens* (Di hua jun)	Nasopharyngeal cancer C666-1 cells	Glucose consumption; ATP generation	Targets the PDK1-PI3K/Akt signaling pathway	[38]

Oleanolic acid	*Ligustrum lucidum* (Nv zhen); *Olea europaea* (Qi dun guo)	Human prostate carcinoma PC-3 cells; human breast cancer MCF-7 cells; human hepatoma HepG2 cells	Aerobic glycolysis; lipid metabolism; mitochondrial OXPHOS	Induces PKM2 to PKM1 switch, inhibits the phosphorylation of mTOR, and PPAR*γ*, decreases expression of c-Myc-dependent hnRNPA1 and hnRNPA1, activates AMPK, and elevates COX-2 expression	[39–41]

Oridonin	*Rabdosia rubescens* (Dong ling cao)	Human colorectal cancer SW480 and SW620 cells; uveal melanoma OCM-1 and MUM2B cells	Fatty acid synthesis; cellular levels of palmitate and stearic acid	Inhibits FAS and SREBP1 mRNA and protein expression	[42, 43]

Osthole	*Cnidium monnieri* (She chuang zi)	Human ovarian cancer SKOV3 cells	Fatty acid synthesis	Inhibits the phosphorylation of Akt and mTOR and downregulates FASN expression	[44]

Prosapogenin A	*Veratrum nigrum* (Li lu)	Breast cancer MCF-7 cells	Glycolysis	Reduces the expression of STAT3, GLUT1, HK, and PFKL mRNA	[45]

Quercetin	Folium Mori (Sangye); Radix Bupleuri (Chai hu)	HepG2 cells; Dalton's lymphoma mice	Fatty acid synthesis; glycolysis	Decreases FASN expression, reduces NADPH levels, and downregulates PI3K-AKT-p53 pathway	[46, 47]

Shikonin	*Lithospermum erythrorhizon* (Zi cao)	Human macrophage U937 cells; human breast cancer SK-BR-3 cells; human promyeloblastic leukemia *HL60* cells	Mitochondrial dysfunction; cellular lactate production; glucose consumption	Targets TrxR1 and inhibits PKM2 activity	[48–50]

Silibinin	*Silybum marianum* (Shui fei ji)	Transgenic adenocarcinoma of the mouse prostate; human colorectal carcinoma SW480 cells	Glucose content and uptake; lactate, citrate, phosphatidylcholine, and cholesterol levels; mitochondrial OXPHOS	Inhibits PIK3CA-AKT-MTOR and activates MAP2K1/2-MAPK1/3 pathways	[51, 52]

Tetrandrine	*Stephania tetrandra* (Fen fang ji)	Prostatic cancer PC-3 cells; renal carcinoma 786-O cells	Glucose uptake	Induces AMPK phosphorylation and OXPHOS impairment	[29]

Wogonin	*Scutellaria baicalensis* (Huang qin)	Resistant human colon cancer HCT116 cells	Glucose uptake; lactate generation	Decreases the expression of HKII, PDHK1, LDHA, and HIF-1*α* and inhibits PI3K/Akt signaling pathway	[53]

EGCG	Green tea (Lv cha)	Human pancreatic cancer MIA PaCa-2 cells; human tongue carcinoma cells; MCF-7 cells; MDA-MB-231 cells; HT-29 cells; A549 cells; LNCaP cells; lung cancer xenografts	Lactate production, anaerobic glycolysis; glucose consumption and uptake; fatty acid metabolism	Inhibits HK2 expression, LDHA activity, FAS activity, and FASN activity; mediates the insulin-response via GLUT1, GLUT4; and GLUT12, and activates AMPK pathway	[54–59]

Oroxylin A	*Oroxylum indicum* (Mu hu die)	HepG2 cells; MDA-MB-231 cells; MCF-7 cells; A549 cells; female athymic BALB/c nude mice	Glucose uptake; lactate generation; ROS accumulation; ATP generation; glycolysis	Suppresses mRNA levels of PDK1, LDHA, and HK II; inhibits HIF-1*α* expression and stability; promotes SOD2 gene expression and SIRT3 activation; inactivates the c-Src/AKT/HK II pathway	[60–63]
